# High-Field Asymmetric Waveform Ion Mobility Spectrometry Interface Enhances Parallel Reaction Monitoring on an Orbitrap Mass Spectrometer

**DOI:** 10.1021/acs.analchem.2c01287

**Published:** 2022-11-08

**Authors:** Weixian Deng, Jihui Sha, Fanglei Xue, Yasaman Jami-Alahmadi, Kathrin Plath, James Wohlschlegel

**Affiliations:** † David Geffen School of Medicine, Department of Biological Chemistry, University of California Los Angeles, Los Angeles, California 90095, United States; ‡ Molecular Biology Interdepartmental Graduate Program, University of California Los Angeles, Los Angeles, California 90095, United States; § University of Technology Sydney, Ultimo, New South Wales 2007, Australia

## Abstract

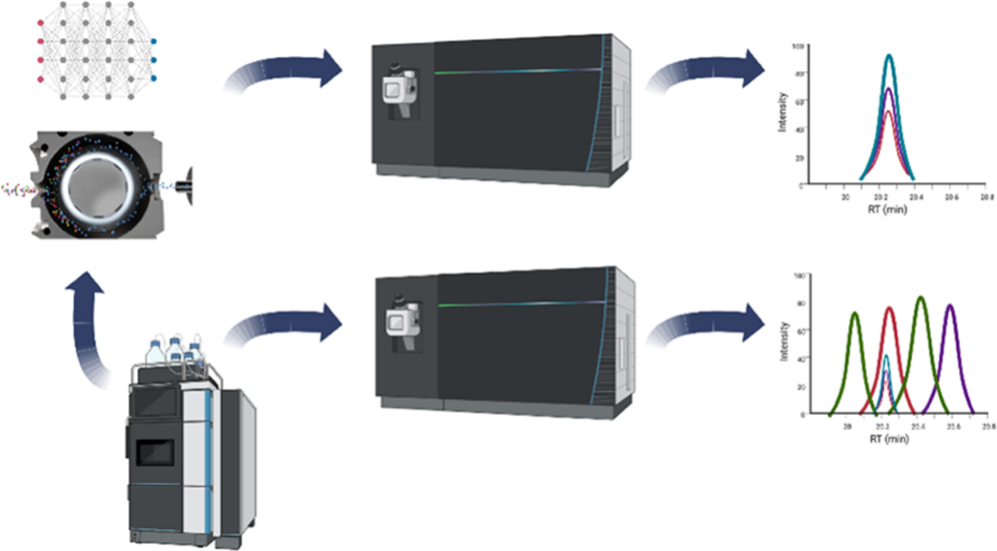

High-field asymmetric waveform ion mobility spectrometry (FAIMS) enables gas-phase separations on a chromatographic time scale and has become a useful tool for proteomic applications. Despite its emerging utility, however, the molecular determinants underlying peptide separation by FAIMS have not been systematically investigated. Here, we characterize peptide transmission in a FAIMS device across a broad range of compensation voltages (CVs) and used machine learning to identify charge state and three-dimensional (3D) electrostatic peptide potential as major contributors to peptide intensity at a given CV. We also demonstrate that the machine learning model can be used to predict optimized CV values for peptides, which significantly improves parallel reaction monitoring workflows. Together, these data provide insight into peptide separation by FAIMS and highlight its utility in targeted proteomic applications.

## Introduction

FAIMS differential ion mobility devices typically function as an ion filter placed between the electrospray ionization (ESI) ion source and the mass spectrometer (MS).^1−3^ As ions move through high and low electric fields of opposite polarity generated by an asymmetric waveform, a small compensation voltage (CV) can be applied to the waveform that enables a subset of ions to travel through the device based on their physicochemical properties while effectively filtering out other ions.^4^ By applying different CVs, FAIMS devices are thereby able to fractionate analytes such as peptides. Gas-phase fractionation by FAIMS is dictated primarily by the ion charge state of the analyte and allows it to effectively remove singly charged interference ions from the desired peptide analytes and simplify the overall analyte composition entering the mass analyzer.^5^ The current commercial FAIMSpro interface being marketed by Thermo Fisher can change CV values in as little as 50 ms, enabling its use on a chromatographic time scale but also requires the development of optimized strategies for alternating CVs and maximizing peptide identification.^5−7^

Despite the advantages of FAIMS, alternating between CVs slows the duty cycle; therefore, only a limited number of CVs can be used in a given experiment without negatively impacting the number of peptide identifications.^8^ This limitation on the number of CVs that can be effectively used in an experiment can lead to peptides being characterized using suboptimal CV settings resulting in compromised sensitivity or a complete failure to detect peptides of interest. A detailed understanding of the determinants that dictate peptide detectability as a function of CV in a FAIMS-based fractionation is still largely lacking but considered essential for maximizing the utility of these analyses. In this study, we analyzed peptide intensity distributions derived from proteomic characterization of the human proteome carried out over a range of different CVs. These analyses identified three-dimensional (3D) electrostatic descriptors as determinants of peptide isoform detectability on FAIMS instruments.

Targeted proteomic methods such as parallel reaction monitoring (PRM) also depend on the selection of optimal CV settings to maximize sensitivity and the lower limit of quantitation (LLOQ) for each target. However, in many cases, the optimal CV for a given peptide target is not empirically known as its selection was based on data sets collected on instruments using generally applied CV settings or not equipped with FAIMS. This uncertainty regarding the ideal CV settings for a given panel of peptides has the potential to greatly diminish the effectiveness of the analysis. In this study, we built a machine learning model to predict a peptide’s peak intensity given its sequence and charge state as a function of CV values and demonstrate its utility in a series of proteomics analyses.

## Experimental Section

### Sample Preparation

HEK293 cells were cultured in high glucose and glycine DMEM containing 10% FBS and 1% penicillin–streptomycin and then harvested by trypsinization and pelleting with 300*g* centrifugation. Cell pellets were lysed by incubation in lysis buffer (8 M urea, 0.1 M tris–HCl pH 8.0) at 4 °C for 30 min followed by centrifugation to clarify the sample. Two milligrams of protein were reduced and alkylated by sequentially incubating with 5 mM TCEP and 10 mM iodoacetamide for 30 min at room temperature in the dark. The protein sample was then diluted 4-fold with 0.1 M tris–HCl pH 8.0 to reduce the final urea concentration to 2 M before incubating overnight at 37 °C with a 50 μg trypsin protease. Peptide digests were desalted using Pierce C18 tips (100 μL bed volume), dried, and then reconstituted in 5% formic acid to make the final concentration to be 0.2 μg/μL and inject 4 μL per injection.

### LC-MS/MS

#### Liquid Chromatography Settings

##### Short Gradient

Tryptic peptide digest (800 ng) prepared from whole cell lysates of HEK293 cells was loaded onto a 25 cm long, 75 μm inner diameter fused-silica capillary, packed in-house with bulk 1.9 μm ReproSil-Pur C18 beads. Peptides were delivered using a Thermo Scientific EASY-nLC 1200 HPLC system. The mobile phase buffers are buffer A (water solution with 3% DMSO and 0.1% formic acid) and buffer B (80% acetonitrile solution with 3% DMSO and 0.1% formic acid). The 70 min short gradient is delivered as follows: 1–6% buffer B from 0 to 6 min at a flow rate of 300 nL/min, 6–28% buffer B from 6 to 54 min at a flow rate of 220 nL/min, 28–32% buffer B from 54 to 62 min at a flow rate of 220 nL/min, and 85% buffer B from 62 to 70 min at a flow rate of 220 nL/min.

##### Long Gradient

The same amount of lysate digest was loaded into the same column. Then, the peptide analyte was eluted using a 140 min gradient of increasing acetonitrile (ACN) as follows: at the start of the gradient, the flow rate is 350 nL/min and 2% buffer B, 2–6% buffer B from 0 to 2 min at a flow rate of 300 nL/min, 2–35% buffer B from 2 to 116 min at a flow rate of 300 nL/min, 35–42% buffer B from 116 to 136 min at a flow rate of 300 nL/min, 42–85% buffer B from 136 to 138 min at a flow rate of 300 nL/min, and 85–95% buffer B from 138 to 140 min at a flow rate of 300 nL/min.

#### Data Acquisition Settings

##### Data-Dependent Acquisition

The eluted peptides were ionized using a Thermo Scientific Nanospray Flex ion source and injected into a Thermo Scientific Orbitrap Eclipse Tribrid mass spectrometer operated in positive mode equipped with a FAIMSpro interface. Spectra were acquired using data-dependent acquisition mode where a 120 k resolution full MS1 scan was followed by sequential MS2 scans at a resolution of 15,000 to utilize the remainder of the 3 s cycle time. MS/MS spectra were collected using a 1.5 *m*/*z* window for precursor ion quadrupole isolation and normalized HCD collision energy of 30% with a dynamic exclusion of 10 s and monoisotopic peak determination set to peptide. The “auto” maximum injection time was selected to allow the orbitrap to calculate the maximum injection time available to maximize sensitivity while maintaining the maximum scan rate. FAIMS separations were performed using FAIMS mode on a standard resolution set to static gas mode with a nitrogen carrier gas flow of 0 L/min and inner and outer electrode temperatures of 100 °C with an asymmetric dispersion voltage (DV) of −5000 V. To selectively filter ions that enter the mass spectrometer, individual compensation voltages (CVs) between the range of −25 and −70 V were applied to sequential survey scans and MS/MS cycles.

##### Parallel Reaction Monitoring

LC method is set the same as DDA experiments. Target precursor ions for different experiments are shown in Supporting Table 1. Precursors are fragmented by HCD at a 30% collision energy and then analyzed using the targeted MS2 mode in Xcalibur at 15,000 resolution on an Orbitrap mass analyzer. PRM experiments were analyzed with Skyline using mProphet models^9^ trained using the second-best peak.

#### Data Analysis

##### Machine Learning

Features for building machine learning models were obtained from the Peptides package. Machine learning models including the linear and stacked ensemble models are built through H2O-AI (3.36.0.1). The empirical constraints used for the stacked ensemble model 2 are generated by ±5 V centered by the prediction reported from stacked ensemble model 1. R code for predicting the optimal CVs from peptide sequences can be found at https://github.com/weixiandeng/FAIMSpredictor.

##### Database Searching

MS/MS database searching was performed using MaxQuant (1.6.10.43) against the human reference proteome from EMBL (UP000005640_9606 HUMAN Homo sapiens, 20874 entries). The search included carbamidomethylation on cysteine as a fixed modification and methionine oxidation and N-terminal acetylation as variable modifications. The digestion mode was set to trypsin and allowed a maximum of two missed cleavages. The precursor mass tolerances were 20 and 4.5 ppm for the first and second searches, respectively, while a 20 ppm mass tolerance was used for fragment ions. Data sets were filtered at a 1% FDR at both the PSM and protein levels. Peptide quantitation was performed using MaxQuant’s LFQ mode with an LFQ minimum ratio count set to 2, the normalization type was set to classic, and fast LFQ was enabled. The MaxQuant peptide intensity and not LFQ intensity was used for all peptide quantitation.

#### Data Availability

The proteomics data are deposited in the MassIVE data repository (https://massive.ucsd.edu) under the identifier MSV000089014.

## Results and Discussion

### Systematic Investigation of Peptide Detectability across Different CV Values

**Figure 1:**
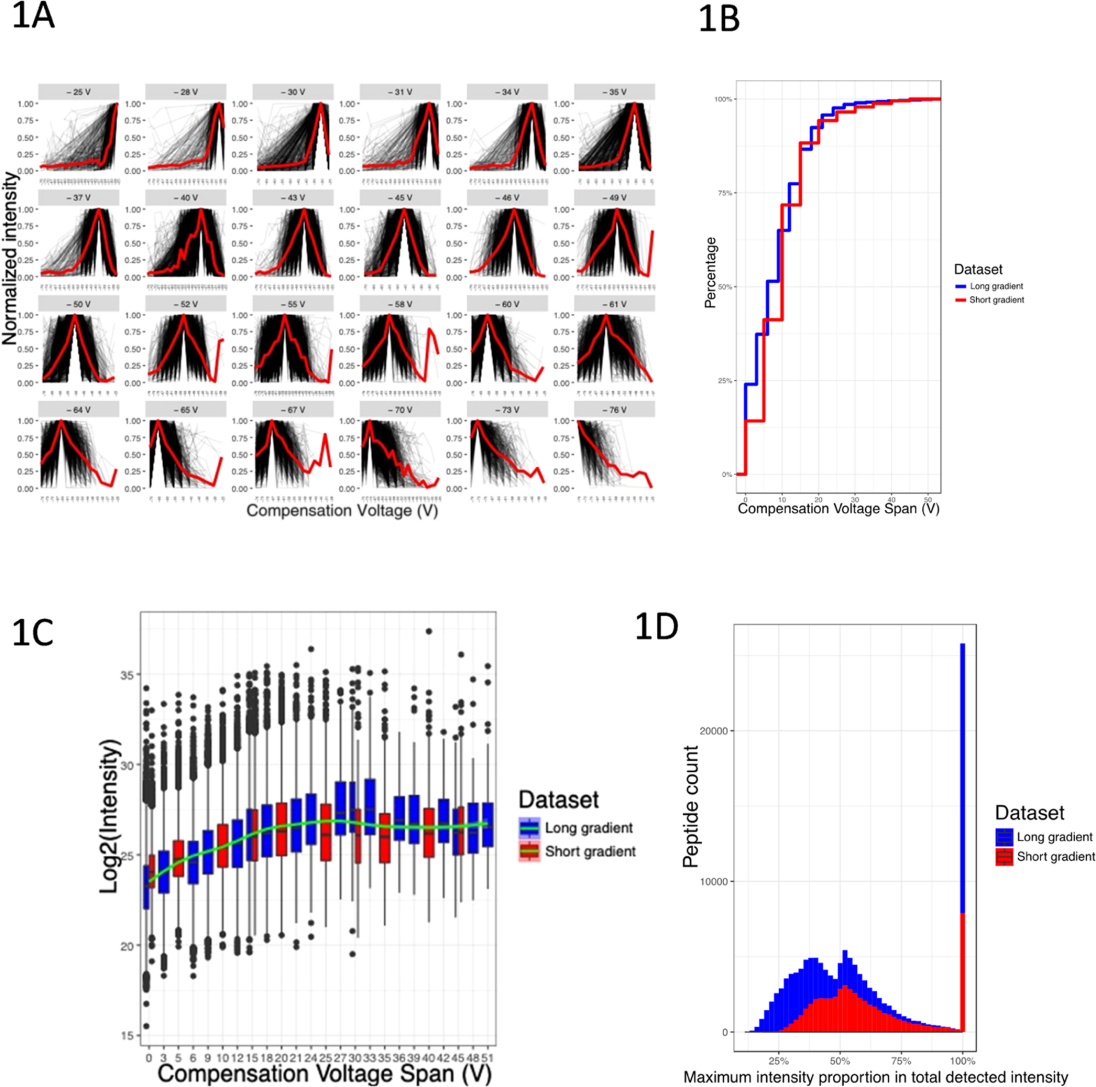
Peptide detectability across different CV values. (A) Normalized intensity distributions for peptides detected in at least three different CV values. Individual peptide intensities were normalized to the maximum observed intensity for that peptide across all CV values to ensure all intensity values are between 0 and 1. The red line connects the median intensities of the detected peptides across CV values. (B) Accumulation curve of the percentage of peptides identified across a given CV span for both the short gradient (red) and long gradient (blue) data sets. (C) Log 2-transformed intensity distribution of peptides identified at different CV values for both the short gradient (red) and long gradient (blue) data sets. The green line indicates the trend. (D) Histogram of the number of peptides binned by the fraction of the peptide intensity found in the highest intensity CV bin relative to the total intensity of that peptide across all bins for the long gradient (blue) and short gradient (red) data sets.

Previous studies have shown that peptide identification can vary widely based on the CV values employed in the analysis.^5−7^ However, peptide detectability has not been systematically investigated as a function of CV. To address this gap, we examined peptide detectability across a range of CVs using two commonly used LC-MS settings. We analyzed HEK293 whole cell lysate peptide digests by LC-MS/MS using data-dependent analysis and label-free quantification with (1) 70 min LC gradients across 9 CV values (−20 to −70 V in −5 V increment) with a single CV per injection and (2) 140 min LC gradients across 18 CV values (−20 to −76 V in −3 V increments) with three alternating CVs per injection (Table S1). To examine the peptide intensity distributions, we first filtered the two data sets to only include peptides detected in at least three different CV values, which included 56.02% of the peptides identified in the short gradient data set and 57.43% of peptides in the long gradient data set. Peptide intensities were normalized to the maximum intensity observed for each peptide across the CV range and then plotted (Figure 1A). Except for the extreme ends of the CV range (−25 and −76 V), the peptide intensities of individual CV values appear as relatively narrow, well-defined bell-shaped distributions. From these data, we observe several key features related to how peptide detectability is influenced by CV. First, we find that almost all peptides are detectable in an approximately 20 V span of CV values centered around the CV value that corresponds to their maximum intensity (94.26% of peptides under 20 V in the short gradient data and 95.74% peptides in the long gradient data) (Figure 1B). Second, we explored the correlation between a peptide’s maximum intensity and its detectable CV span (Figure 1C) and observed a clear trend in which peptides with a higher detectable intensity exhibit wider CV spans. Finally, we asked how necessary it is to know a peptide’s ideal CV to detect it at its highest intensity. Therefore, we measured the percentage of the intensity for each peptide found in its most intense CV bin relative to its total intensity across all CV values. As shown in Figure 1D, the peptide intensity in the most intense CV bin accounts for over 50% of the total detectable intensity for a large fraction of identified peptides (47.1% for the long gradient, 66.1% for the short gradient), suggesting that maximizing the sensitivity of detection for any given peptide will require sampling of that peptide at its optimized CV value. From these data, we conclude that many of the peptides observed in a standard proteomic analysis are detectable only in a relatively narrow CV range and that approaches to maximizing sensitivity will require the integration of this knowledge into data acquisition strategies.

### Linear Model Suggests Main Determination Factors for Peptide’s Peak Intensity CV

Previous reports^5^ have shown that a peptide isoform’s ability to pass through the FAIMS device at a given CV value can be weakly correlated with the charge state. However, the identification of other physicochemical properties displaying a clear correlation with FAIMS transmission is only now emerging.^1,2,10,11^ Using a machine learning approach, we attempted to identify additional determinants that govern peptide transmission by FAIMS. We built a linear regression model to identify factors that impact peptide detectability and then used Lasso regression to minimize the number of factors in the model.

**Figure 2:**
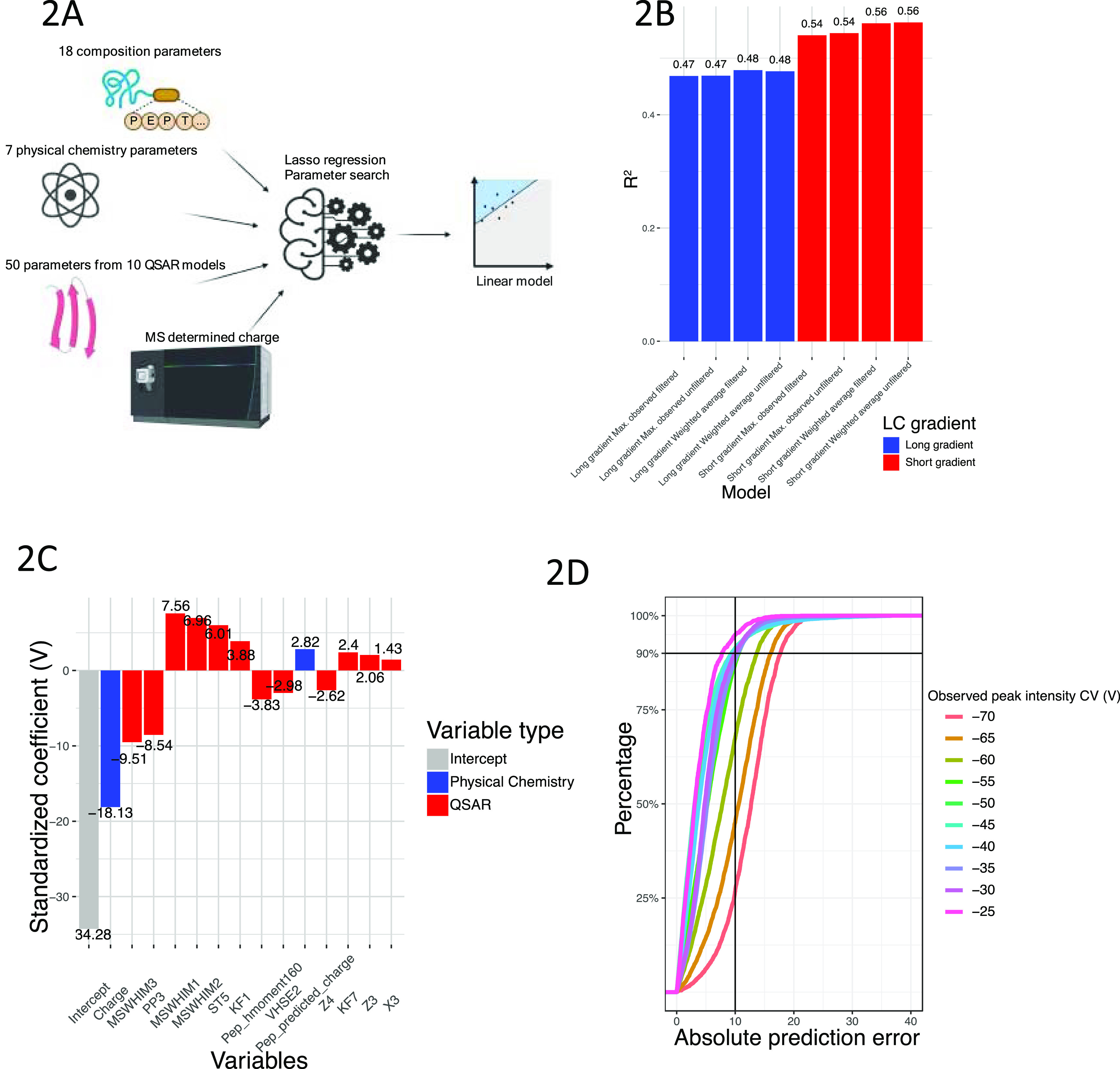
Linear model suggests main determination factors for peptide’s peak intensity CV. (A) Schematic of the machine learning approach for building the linear model. (B) *R*^2^ of the eight different linear models built with (1) different data sets, (2) filtered by only observed under one CV value or not, and (3) either the observed intensity or weighted average peak intensity CV. (C) Standardized coefficients for the top 15 parameters of the model built by short gradient data set after filtering out single observations and using CV corresponding to the highest weighted average peak intensity. (D) Accumulation curves of the percentage of peptides with a given absolute CV prediction error. Each line corresponds to the peptides whose maximum weighted average intensity was observed at the given CV.

The goal of the model was to use peptide sequence and charge state to predict the CV value where a given peptide can be detected at maximum intensity. We first built a pool of parameters for each peptide isoform. In addition to the observed charge state from MS, we calculated 76 different parameters for each peptide comprised of peptide charge, 18 peptide amino acid composition features, 7 basic physicochemical properties,^12−14^ and 10 quantitative structure–activity relationship (QSAR)^15−21^ descriptors derived from 50 peptide sequence features for a total of 76 parameters for each peptide (Figure 2A). Since the data used to build the model were acquired under discrete CV values and could potentially miss the CV corresponding to the maximal intensity using our approach, we calculated the weighted average CV values for the peak in addition to the observed CV value corresponding to the maximum peak intensity. For peptides detected at only a single CV value, we tested the effects of excluding or including them in the model. We then trained the Lasso regression using either the weighted average CV or the observed CV corresponding to the highest peak intensity with or without the inclusion of the single observation data using the two data sets quantified under different LC gradients (Figure 2A) for a total of eight different trained linear models (Table S2).

As shown in Figure 2B, models trained from both short and long gradient data sets show a linear correlation between peptide parameters and weighted average CV of peak intensity/observed peak intensity CV, with the short gradient data showing overall better linearity. Additionally, filtering out single observation data resulted in slightly better prediction accuracy across training sets. The model showing the highest *R*^2^ (0.56) and the lowest mean absolute error MAE (5.89) was trained with the short gradient data set and lacking the single observation data (Figure 2C). From this model, we observed that the charge state is the highest contributor across all used parameters consistent with previous studies.^5,22^ Interestingly, we see MS-WHIM1 and MS-WHIM3, which are both descriptors for the 3D molecule surface electrostatic potential,^23,24^ ranked second and third, suggesting that charge distribution across the 3D structure of peptides significantly contributes to efficient FAIMS transmission. In addition, PP3, peptide predicted charge when pH = 7, the Cruciani property H-bonding descriptor, and other QSAR features also contributed to the model. It is also worth noting that a recent study by Sinn et al.^10^ examined the factors contributing to the FAIMS-based separation of cross-linked peptides and identified a similar collection of determinants.

Next, we evaluated the quality of the model’s prediction by examining peak peptide intensities observed at different CV values. As shown in Figure 2D, we predicted CVs corresponding to the highest weighted average peak intensity for all peptides from the short gradient data set lacking the single observation data and then calculated the absolute difference between the CVs predicted from the model and their corresponding empirically observed CV values. These data were used to build an accumulation curve to determine the proportion of peptides that exist below a given difference in CV values. Peak intensities determined for CV settings less negative than −55 V are generally of high quality, with ∼90% of peptide isoforms having an absolute error of less than 10 V. However, when the peak intensity CV was more negative than −55 V, the linear model prediction quality diminished quickly, suggesting that other as of yet unaccounted for factors influence FAIMS transmission at the low end of the CV range.

### Deep Learning Model Predicts Peptide’s Best CV Value for Improving PRM Performance

Targeted proteomic techniques such as parallel reaction monitoring have emerged as powerful analytical tools for monitoring the abundance of discrete sets of peptides in a high-throughput and sensitive manner. Although the utility of FAIMS in targeted proteomic workflows may offer significant advantages with respect to sensitivity, the integration of these technologies has not been widely reported. Based on the observations by our group and Hebert et al.^22^ that peptide intensity peaks within a relatively narrow CV range, we hypothesized that targeted proteomic assays would require accurate prediction of a peptide’s optimal CV value to maximize the benefit from FAIMS-based analyses. However, the 5.89 V MAE obtained from the linear model built above may not be sufficient. Therefore, we trained two additional machine learning models that can provide higher accuracy at the expense of knowing the relative contribution from different determinants.

**Figure 3:**
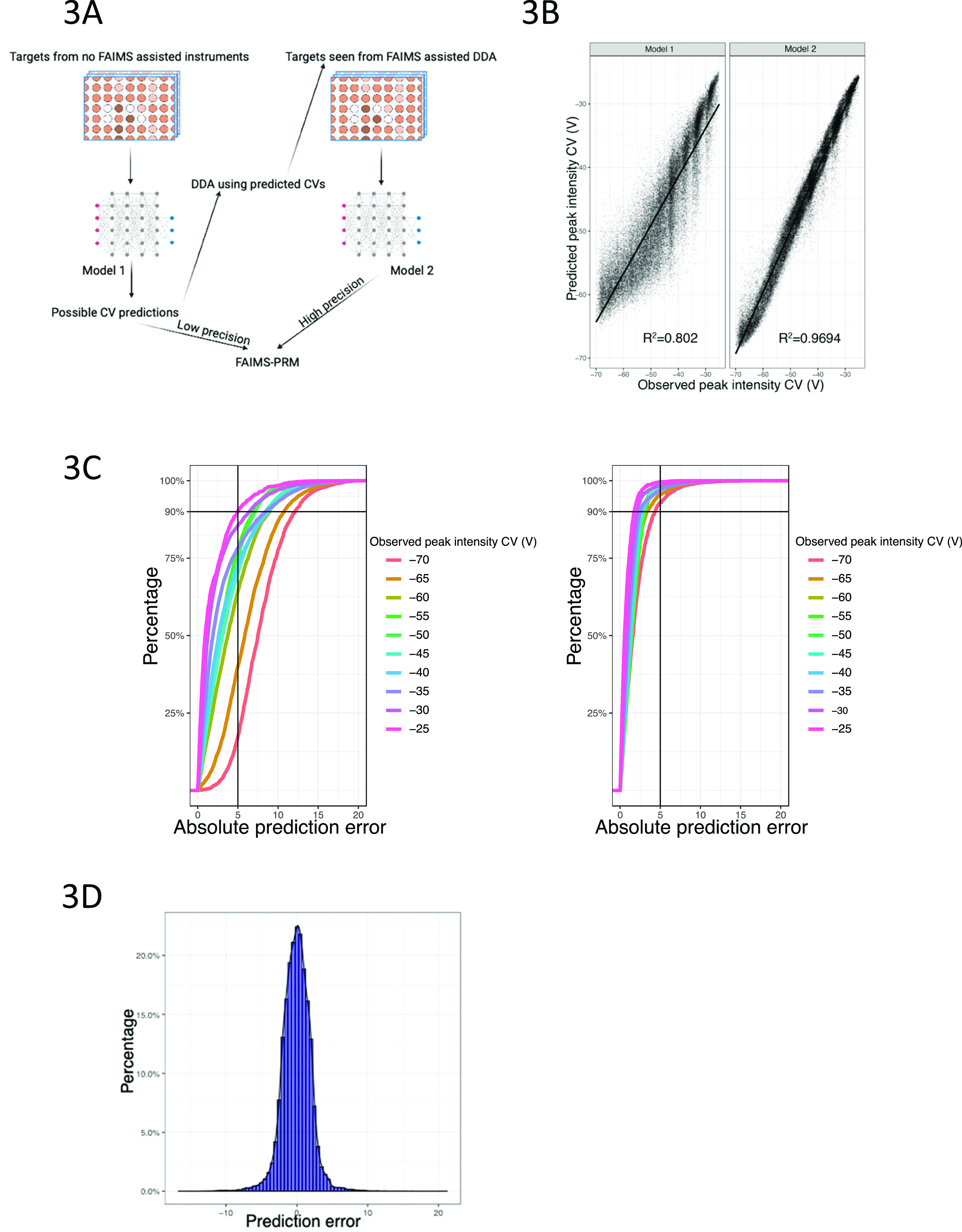
Higher-order machine learning models predict CV corresponding to the maximum peptide peak intensity. (A) Scheme for using stacked ensemble models to predict the peptide peak intensity at different CV values. (B) Correlation of model 1 and 2′s CV corresponding to the predicted highest peak intensity, with the observed CV corresponding to the measured highest weighted average peak intensity. (C) Accumulation curves of the percentage of peptides with a given absolute CV prediction error as predicted by model 1 (left) and model 2 (right). Each line corresponds to the peptides whose maximum weighted average intensity was observed at the given CV. (D) Prediction error histogram for all peptides in the short gradient data set.

We first trained a stacked ensemble model (model 1) using the same 76 features described above for the linear model, which resulted in an overall MAE of 3.81 V for the total set of data (Figure 3B, left). However, we noticed that for peptides with a weighted average peak intensity CV more negative than −55 V, the prediction error increased significantly (Figure 3C, left). Therefore, we examined whether prediction could be improved by the inclusion of an additional constraint where we included an empirically determined CV value at which the given peptide was detected irrespective of whether this was the CV where the maximum intensity is observed. Essentially, based on the prediction error in model 1, we take one of possible three CV values (the predicted CV from model 1 and 5 V above and below it) corresponding to the CV closest to the observed peak intensity and use it as an empirical constraint to train model 2. As shown in Figure 3C, right, this approach generated a more accurate model with lower prediction errors but similar performance in all observed peak intensity CV bins.

To optimize CV usage in the context of PRM experiments, we propose to integrate these two machine learning models into a single workflow (Figure 3A). Researchers commonly select peptides for inclusion in a PRM target list based on experimental data (DDA or PRM/SRM) collected on instruments not equipped with FAIMS or predicted peptide sequences lacking any empirical data supporting their identification. For these peptides, we utilize the peptide sequence and charge state to predict the CV using model 1. In this case, model 1 will generate a low precision CV prediction that can either be (1) used directly in a targeted assay or (2) used as a starting point for additional experiments to narrow down its optima CV. In the latter case, a 3-alternating-CV run (DDA with inclusion list or PRM) using the predicted CV value as well as CV values shifted 5 V in either direction to determine the detectability of the peptide of interest. If the peptide is detected, then its empirically determined CV value (even if not optimized) can be used as a constraint in model 2 to predict higher precision CV values for future targeted assays. We simulated the process on the short gradient data where over 92% of peptides can be covered by the three CV values used in the pilot experiment, and the overall MAE for the high precision prediction is 1.48 V. The prediction error for each model for a given observed peak intensity CV bin is shown in Figure 3C, while Figure 3D shows the prediction error for the whole short gradient data set.

### FAIMS Improves PRM Performance

**Figure 4:**
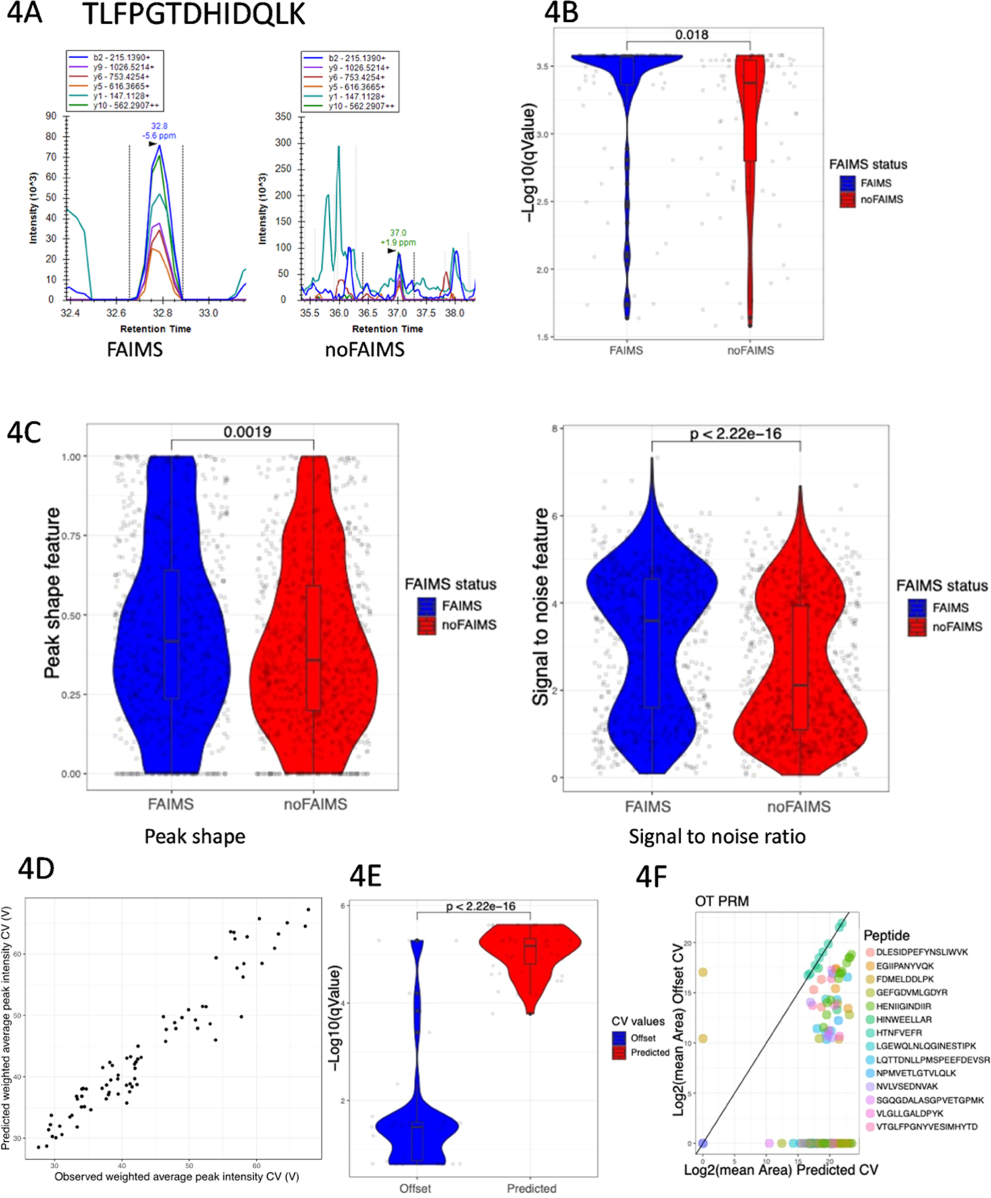
FAIMS and suitable CV settings improve PRM performance. (A) XIC of the peptide: TLFPGTDHIDQLK acquired with and without FAIMS enabled. (B) mProphet *q* value distribution (−log 10 transformed) for all of the target peptides analyzed using parallel reaction monitoring with and without FAIMS. (C) Distribution of peak shape scores (left) and signal-to-noise ratio score (right) for all extracted fragment ion transitions from PRM data acquired with (blue) and without (red) FAIMS. (D) Observed CV vs predicted CV corresponding to the highest weighted average peak intensity for PRM target peptides. (E) Distribution of the −log 10 (mProphet *q*-values) for PRM target peptides using the CV values either predicted by machine learning model (red) or offset by +10 V from the predicted CV (blue). (F) Scatter plot of the mean area of fragment ion transitions from PRM target peptides where data were acquired using the predicted CV (*x*-axis) and the predicted CV offset by +10 V (*y*-axis).

We next examined the significance of FAIMS-based separations on targeted PRM experiments as well as tested the utility of our CV prediction models. First, we tested the performance of PRMs in the presence and absence of a FAIMS device using an Orbitrap (OT) mass analyzer, as implemented on the Thermo Fisher Fusion Eclipse mass spectrometer to explore the extent to which FAIMS might improve PRM analyses. We selected 85 peptide isoforms whose maximal peak intensity occurred at a range of different CV values as targets for the experiment. Representative extracted ion chromatograms are shown in Figure 4A and demonstrate that FAIMS can significantly improve PRM data quality. To quantify the performance improvement provided by FAIMS, we built mProphet models and compared *q*-values for target peptides between FAIMS and no FAIMS data sets. The use of a FAIMS device significantly boosted *q*-values highlighting the improved data quality (*p*-value = 0.018) (Figure 4B). To further characterize this improvement, we examined individual mProphet features at the fragment ion transition level and found that the peak shape and signal-to-noise ratio are significantly higher for FAIMS vs no FAIMS with *p*-values of 0.0019 and 2.2e-16, respectively (Figure 4C). These data suggest that the use of a FAIMS device significantly improves PRM analyses.

We next explored the importance of selecting accurate CVs in a PRM analysis. To this end, we utilized our machine learning model to build a predictor for peptide CV based on its CV and applied it to the peptide panel described in Figure 3. Figure 4D shows a comparison between the empirically determined and predicted CV values for these peptides. These data validate the predictor and demonstrate its ability to accurately predict CV values based on peptide sequence. Next, we performed a parallel reaction monitoring experiment targeting this peptide panel using FAIMS with both the predicted CV and a CV value shifted by 10 V (Tables S3 and S4). We reasoned that if accurate CV settings were necessary for an optimized PRM experiment, then the data set with the predicted CV values should be significantly better than that with the shifted less optimal CV values. The mProphet *q* value distribution for both the predicted CV and shifted CV data sets is shown in Figure 4E and shows that the data quality of target peptides is significantly increased when data are acquired using the predicted CV values. This observation is also clearly observed in scatterplots comparing the intensity of individual peptide fragment ion transitions between predicted and shifted CV experiments (Figure 4F). Together, these data emphasize the importance of acquiring data using optimized CV values and that this process can be facilitated using the CV predictor developed based on our machine learning model.

## Conclusions

In this study, we systematically profiled the intensity distribution of peptides during FAIMS separation across different CV settings and found that the majority of peptides are efficiently transmitted through relatively narrow CV windows. Using machine learning approaches, we showed that the charge state together with 3D descriptors such as MS-WHIM scores is the top determinant for dictating peptide isoform transmission across CV space and that this information can be used to accurately predict optimal peptide CV from a peptide’s sequence. Furthermore, we find that the proper selection of CV values is essential for PRM assay data quality with an optimized CV setting leading to significant improvements in detection and quantitation.

## Supporting Information Available

CV settings for MS runs (Table S1); machine learning model data sets (Table S2); PRM target list (Table S3); and offset PRM target list (Table S4) (PDF)

## References

[ref1] Guevremont R. High-Field Asymmetric Waveform Ion Mobility Spectrometry: A New Tool for Mass Spectrometry. J. Chromatogr. A 2004, 1058, 3–19. 10.1016/S0021-9673(04)01478-5. 15595648

[ref2] Purves R. W.; Guevremont R. Electrospray Ionization High-Field Asymmetric Waveform Ion Mobility Spectrometry–Mass Spectrometry. Anal. Chem. 1999, 71, 2346–2357. 10.1021/ac981380y. 21662783

[ref3] Dodds J. N.; Baker E. S. Ion Mobility Spectrometry: Fundamental Concepts, Instrumentation, Applications, and the Road Ahead. J. Am. Soc. Mass. Spectrom. 2019, 30, 2185–2195. 10.1007/s13361-019-02288-2. 31493234 PMC6832852

[ref4] Barnett D. A.; Ells B.; Guevremont R.; Purves R. W. Application of ESI-FAIMS-MS to the Analysis of Tryptic Peptides. J. Am. Soc. Mass. Spectrom. 2002, 13, 1282–1291. 10.1016/s1044-0305(02)00527-5. 12443018

[ref5] Pfammatter S.; Bonneil E.; McManus F. P.; Prasad S.; Bailey D. J.; Belford M.; Dunyach J. J.; Thibault P. A Novel Differential Ion Mobility Device Expands the Depth of Proteome Coverage and the Sensitivity of Multiplex Proteomic Measurements. Mol. Cell. Proteomics 2018, 17, 2051–2067. 10.1074/mcp.tir118.000862. 30007914 PMC6166672

[ref6] Bekker-Jensen D. B.; Martínez-Val A.; Steigerwald S.; Rüther P.; Fort K. L.; Arrey T. N.; Harder A.; Makarov A.; Olsen J. V. A Compact Quadrupole-Orbitrap Mass Spectrometer with FAIMS Interface Improves Proteome Coverage in Short LC Gradients. Mol. Cell. Proteomics 2020, 19, 716–729. 10.1074/mcp.tir119.001906. 32051234 PMC7124470

[ref7] Schweppe D. K.; Prasad S.; Belford M. W.; Navarrete-Perea J.; Bailey D. J.; Huguet R.; Jedrychowski M. P.; Rad R.; McAlister G.; Abbatiello S. E.; Woulters E. R.; Zabrouskov V.; Dunyach J. J.; Paulo J. A.; Gygi S. P. Characterization and Optimization of Multiplexed Quantitative Analyses Using High-Field Asymmetric-Waveform Ion Mobility Mass Spectrometry. Anal. Chem. 2019, 91, 4010–4016. 10.1021/acs.analchem.8b05399. 30672687 PMC6993951

[ref8] Hebert A. S.; Prasad S.; Belford M. W.; Bailey D. J.; McAlister G. C.; Abbatiello S. E.; Huguet R.; Wouters E. R.; Dunyach J. J.; Brademan D. R.; Westphall M. S.; Coon J. J. Comprehensive Single-Shot Proteomics with FAIMS on a Hybrid Orbitrap Mass Spectrometer. Anal. Chem. 2018, 90, 9529–9537. 10.1021/acs.analchem.8b02233. 29969236 PMC6145172

[ref9] Reiter L.; Rinner O.; Picotti P.; Hüttenhain R.; Beck M.; Brusniak M.-Y.; Hengartner M. O.; Aebersold R. MProphet: Automated Data Processing and Statistical Validation for Large-Scale SRM Experiments. Nat. Methods 2011, 8, 430–435. 10.1038/nmeth.1584. 21423193

[ref10] Sinn L. R.; Giese S. H.; Stuiver M.; Rappsilber J. Leveraging Parameter Dependencies in High-Field Asymmetric Waveform Ion-Mobility Spectrometry and Size Exclusion Chromatography for Proteome-Wide Cross-Linking Mass Spectrometry. Anal. Chem. 2022, 94, 4627–4634. 10.1021/acs.analchem.1c04373. 35276035 PMC8943524

[ref11] Purves R. W.; Barnett D. A.; Guevremont R. Separation of Protein Conformers Using Electrospray-High Field Asymmetric Waveform Ion Mobility Spectrometry-Mass Spectrometry. Int. J. Mass Spectrom. 2000, 197, 163–177. 10.1016/s1387-3806(99)00240-7.

[ref12] Bjellqvist B.; Hughes G. J.; Pasquali C.; Paquet N.; Ravier F.; Sanchez J.; Frutiger S.; Hochstrasser D. The Focusing Positions of Polypeptides in Immobilized PH Gradients Can Be Predicted from Their Amino Acid Sequences. Electrophoresis 1993, 14, 1023–1031. 10.1002/elps.11501401163. 8125050

[ref13] Juretić D.; Lučić B.; Zucić D.; Trinajstić N. Protein Transmembrane Structure: Recognition and Prediction by Using Hydrophobicity Scales through Preference Functions. Theor. Comput. Chem. 1998, 5, 405–445. 10.1016/s1380-7323(98)80015-0.

[ref14] Guruprasad K.; Reddy B. V. B.; Pandit M. W. Correlation between Stability of a Protein and Its Dipeptide Composition: A Novel Approach for Predicting in Vivo Stability of a Protein from Its Primary Sequence. Protein Eng., Des. Sel. 1990, 4, 155–161. 10.1093/protein/4.2.155. 2075190

[ref15] Cruciani G.; Baroni M.; Carosati E.; Clementi M.; Valigi R.; Clementi S. Peptide Studies by Means of Principal Properties of Amino Acids Derived from MIF Descriptors. J. Chemom. 2004, 18, 146–155. 10.1002/cem.856.

[ref16] Liang G.; Li Z. Factor Analysis Scale of Generalized Amino Acid Information as the Source of a New Set of Descriptors for Elucidating the Structure and Activity Relationships of Cationic Antimicrobial Peptides. QSAR Comb. Sci. 2007, 26, 754–763. 10.1002/qsar.200630145.

[ref17] Eisenberg D.; Weiss R. M.; Terwilliger T. C. The Hydrophobic Moment Detects Periodicity in Protein Hydrophobicity. Proc. Natl. Acad. Sci. U.S.A. 1984, 81, 140–144. 10.1073/pnas.81.1.140. 6582470 PMC344626

[ref18] Yang L.; Shu M.; Ma K.; Mei H.; Jiang Y.; Li Z. ST-Scale as a Novel Amino Acid Descriptor and Its Application in QSAM of Peptides and Analogues. Amino Acids 2010, 38, 805–816. 10.1007/s00726-009-0287-y. 19373543

[ref19] Tian F.; Zhou P.; Li Z. T-Scale as a Novel Vector of Topological Descriptors for Amino Acids and Its Application in QSARs of Peptides. J. Mol. Struct. 2007, 830, 106–115. 10.1016/j.molstruc.2006.07.004.

[ref20] Mei H.; Liao Z. H.; Zhou Y.; Li S. Z. A New Set of Amino Acid Descriptors and Its Application in Peptide QSARs. Biopolymers 2005, 80, 775–786. 10.1002/bip.20296. 15895431

[ref21] Sandberg M.; Eriksson L.; Jonsson J.; Sjöström M.; Wold S. New Chemical Descriptors Relevant for the Design of Biologically Active Peptides. A Multivariate Characterization of 87 Amino Acids. J. Med. Chem. 1998, 41, 2481–2491. 10.1021/jm9700575. 9651153

[ref22] Hebert A. S.; Prasad S.; Belford M. W.; Bailey D. J.; McAlister G. C.; Abbatiello S. E.; Huguet R.; Wouters E. R.; Dunyach J.-J.; Brademan D. R.; Westphall M. S.; Coon J. J. Comprehensive Single-Shot Proteomics with FAIMS on a Hybrid Orbitrap Mass Spectrometer. Anal. Chem. 2018, 90, 9529–9537. 10.1021/acs.analchem.8b02233. 29969236 PMC6145172

[ref23] Zaliani A.; Gancia E. MS-WHIM Scores for Amino Acids: A New 3D-Description for Peptide QSAR and QSPR Studies. J. Chem. Inf. Comput. Sci. 1999, 39, 525–533. 10.1021/ci980211b.

[ref24] Zaliani A.; Gancia E. QSPR Studies. J. Chem. Inf. Comput. Sci. 1999, 525–533. 10.1021/ci980211b.

