# Supplementary material for: High-Field Asymmetric Waveform Ion Mobility Spectrometry Interface Enhances Parallel Reaction Monitoring on an Orbitrap Mass Spectrometer
Source: Anal Chem. 2022 Nov 8;94(46):15939–47. doi: 10.1021/acs.analchem.2c01287 (PMC9685594; doi:10.1021/acs.analchem.2c01287)
Supplement: Supplementary file 1 — ac2c01287_si_001.pdf [file ac2c01287_si_001.pdf]

Supporting Information

High-field asymmetric waveform ion mobility spectrometry interface enhances parallel reaction monitoring on an Orbitrap mass spectrometer

Weixian Deng[1,2,*], Jihui Sha[1,*], Fanglei Xue[3], Yasaman Jami-Alahmadi[1], Kathrin Plath[1], James Wohlschlegel[1#]

[1]David Geffen School of Medicine, Department of Biological Chemistry, University of California Los Angeles, Los Angeles, CA 90095, USA

[2]Molecular Biology Interdepartmental Graduate Program, University of California Los Angeles, Los Angeles, CA 90095, USA

[3]University of Technology Sydney, Ultimo NSW 2007, Australia

*denotes equal contribution

#Corresponding author: James Wohlschlegel (jwohl@mednet.ucla.edu)

SUPPORTING INFORMATION

Table S1. CV settings for MS Runs

Table S2. Machine Learning Model Datasets

Table S3. PRM Target List

Table S4. Offset PRM Target List

Table S1: CV settings for MS Runs

Injection No.	Single CV, 70 min/run (V)	3 CV, 140 min/run (V)
1	-25	-25, -28, -31
2	-30	-34, -37, -40
3	-35	-43, -46, -49
4	-40	-53, -55, -58
5	-45	-61, -64, -67
6	-50	-70, -73, -76
7	-55	
8	-65	
9	-70	

Table S2: Machine Learning Model Datasets

Model	Dataset	Single observation filtered	Weighted average	Measured
1	Short gradient	+	+	
2	Short gradient	+		+
3	Short gradient		+	
4	Short gradient	+		+
5	Long gradient	+	+	
6	Long gradient	+		+
7	Long gradient		+	
8	Long gradient	+		+

Table S3. PRM Target List

Peptide	Precursor Mz	Precursor Charge	CV
DLESIDPEFYNSLIWVK	1034.517313	2	30
DLTQAWDLYYHVFR	913.946661	2	30
LAPPLVTLLSGEPEVQYVALR	1133.146286	2	30
LGEWQLNLQGINESTIPK	1020.541654	2	30
LQTTDNLLPMSPEEFDEVSR	1161.051922	2	30
NDATAQAFLAEASVMTQLR	1019.007121	2	30
NPMVETLGTVLQLK	771.931634	2	30
NVFDEAILAALEPPEPK	926.988191	2	30
VTGLFPGNYVESIMHYTD	1021.98004	2	30
FDSLTDLVEHYK	733.861731	2	35
FEELFPDWIFPSESER	1014.472905	2	35
FNILGTHTK	515.787641	2	35
GPTPAILESLISINNK	833.972344	2	35
HSNLVQLLGVIVEEK	839.480336	2	35
IQSIAPSLQVITSK	742.937773	2	35
LLYPPETGLFLVR	759.439952	2	35
LQQETAELEESVESGK	888.928528	2	35
LWVIFPGEEGLDYGGVAR	989.507083	2	35
NSFGVIPSTPLAIHTPLMPNQSIDVSLPLNTLGPVMK	1300.365161	3	35
NYLLSLPHK	542.811116	2	35
QFLAPWIESQDWAYAASK	1056.015272	2	35
SPSSEVWFDRR	683.330933	2	35
VMAAENIPENPLK	713.373951	2	35
VYENVGLMQQQK	718.863751	2	35
VYTVVDEMFLAGEIR	871.445105	2	35
WYMQFDDDEK	688.77681	2	35
DIPNENELQFQIK	794.404294	2	40
EAPEPMELDGPK	656.80811	2	40
EGIIPANYVQK	616.337695	2	40
ELDTLNNEIVDLQR	836.431041	2	40
FDMELDDLPK	611.786646	2	40
FIIDEELFGQTHQHELK	695.353094	3	40
GLEISGTFTHR	609.317294	2	40
GYTLADEEEDPLIYQHR	1024.981626	2	45
HINWEELLAR	640.840936	2	40
IEVEQALAHPYLEQYYDPSDEPIAEAPFK	1121.541628	3	40
IQNTGDYYDLYGGEK	868.394124	2	40
KFNILGTNTK	568.32713	2	40
LLGPNASPDGLIPWTR	853.964854	2	40

LLVVGITDPDPDIR	761.927405	2	40
LSRPFQSIIHAK	466.273499	3	40
LTNGIWILAELR	699.908819	2	40
NIIGSSPVADFSAIK	759.911755	2	40
NILPFDHTR	556.795997	2	40
SGQGDALASGPVETGPMK	851.409251	2	40
TSEVQDLQDEVQR	773.870816	2	40
TVWQYHFR	568.785433	2	40
TWPDHGVPSDPGGVLDFLEEVHHK	667.825217	4	40
VMEGTVAAQDEFYR	808.37468	2	40
VNYVVQEAIVVIR	751.440483	2	40
VVLHDGDPNEPVSDYINANIIMPEFETK	1052.844653	3	40
EGFYLFPDGR	600.787838	2	50
ELSAVTFPDIIR	680.877184	2	50
FSPGDFWGR	534.748516	2	50
GEFGDVMLGDYR	679.805902	2	50
HENIIGINDIIR	703.891157	2	50
HTNFVEFR	525.26179	2	50
IWDLADTDGK	567.277303	2	50
LVGTPGAELLK	549.331881	2	50
NVLVSEDNVAK	594.316959	2	50
TLTDEELADWK	660.819532	2	50
VLGLLGALDPYK	629.873913	2	50
LHVSTINLQK	576.840405	2	55
LLQTAATAAQQGGQANHPTAAVVTEK	859.452419	3	55
SGLPDLILGK	506.805499	2	55
WFHPNITGVEAENLLLTR	704.040851	3	55
ELIFEETAR	554.287671	2	60
FLEQVHQLYDDSFPMEIR	756.364894	3	30
FSLENNFLLQHNIR	582.30916	3	60
GQVFDVGPR	487.756341	2	60
HGSFLVR	408.229762	2	60
IPLENLQIIR	604.871705	2	60
LHDINAQMVEDQGFLDSLR	734.360032	3	60
TLFPGTDHIDQLK	495.596307	3	60
VSDFGLTK	433.734543	2	60
YLEQLHQLYSDSFPMELR	757.036778	3	60
FLQESNVLYQHNLR	587.640799	3	60
HAAVLVLR	439.782162	2	65
LGAGPGDAGEVQAHPFFR	609.304186	3	65
TLGSFEFEGHSLTQFVR	652.326768	3	65

WTAPEALR	472.253434	2	65
IADFGWSVHAPSSR	510.588035	3	70
LTQLGTFEDHFLSLQR	635.667013	3	70
QVFLATWK	496.781827	2	70
VADPDHDHTGFLTEYVATR	715.339625	3	70

Table S4. Offset PRM Target List

Peptide	Precursor Mz	Precursor Charge	Predicted CV	offset CV
NPMVETLGTVLQLK	771.931634	2	34	44
LGEWQLNLQGINESTIPK	1020.541654	2	32	42
LQTTDNLLPMSPEEFDEVSR	1161.051922	2	29	39
DLESIDPEFYNSLIWVK	1034.517313	2	31	41
VTGLFPGNYVESIMHYTD	1021.98004	2	30	40
FDMELDDLPK	611.786646	2	43	53
EGIIPANYVQK	616.337695	2	43	53
HINWEELLAR	640.840936	2	43	53
SGQGDALASGPVETGPMK	851.409251	2	38	48
NVLVSEDNVAK	594.316959	2	46	56
HTNFVEFR	525.26179	2	46	56
VLGLLGALDPYK	629.873913	2	48	58
GEFGDVMLGDYR	679.805902	2	48	58
HENIIGINDIIR	703.891157	2	51	61
